# A hierarchical model for estimating the spatial distribution and abundance of animals detected by continuous-time recorders

**DOI:** 10.1371/journal.pone.0176966

**Published:** 2017-05-17

**Authors:** Robert M. Dorazio, K. Ullas Karanth

**Affiliations:** 1 Wetland and Aquatic Research Center, U.S. Geological Survey, Gainesville, Florida, United States of America; 2 Wildlife Conservation Society, Bronx, New York, United States of America; 3 National Centre for Biological Sciences, Bangalore, Karnataka, India; 4 Centre for Wildlife Studies, Bangalore, Karnataka, India; Universitat Zurich, SWITZERLAND

## Abstract

**Motivation:**

Several spatial capture-recapture (SCR) models have been developed to estimate animal abundance by analyzing the detections of individuals in a spatial array of traps. Most of these models do not use the actual dates and times of detection, even though this information is readily available when using continuous-time recorders, such as microphones or motion-activated cameras. Instead most SCR models either partition the period of trap operation into a set of subjectively chosen discrete intervals and ignore multiple detections of the same individual within each interval, or they simply use the frequency of detections during the period of trap operation and ignore the observed times of detection. Both practices make inefficient use of potentially important information in the data.

**Model and data analysis:**

We developed a hierarchical SCR model to estimate the spatial distribution and abundance of animals detected with continuous-time recorders. Our model includes two kinds of point processes: a spatial process to specify the distribution of latent activity centers of individuals within the region of sampling and a temporal process to specify temporal patterns in the detections of individuals. We illustrated this SCR model by analyzing spatial and temporal patterns evident in the camera-trap detections of tigers living in and around the Nagarahole Tiger Reserve in India. We also conducted a simulation study to examine the performance of our model when analyzing data sets of greater complexity than the tiger data.

**Benefits:**

Our approach provides three important benefits: First, it exploits *all* of the information in SCR data obtained using continuous-time recorders. Second, it is sufficiently versatile to allow the effects of both space use and behavior of animals to be specified as functions of covariates that vary over space and time. Third, it allows both the spatial distribution and abundance of individuals to be estimated, effectively providing a species distribution model, even in cases where spatial covariates of abundance are unknown or unavailable. We illustrated these benefits in the analysis of our data, which allowed us to quantify differences between nocturnal and diurnal activities of tigers and to estimate their spatial distribution and abundance across the study area. Our continuous-time SCR model allows an analyst to specify many of the ecological processes thought to be involved in the distribution, movement, and behavior of animals detected in a spatial trapping array of continuous-time recorders. We plan to extend this model to estimate the population dynamics of animals detected during multiple years of SCR surveys.

## Introduction

Spatial capture-recapture (SCR) models provide a statistical framework for estimating the density of individuals in a population, that is, the number of individuals per unit area in a finite region of two-dimensional, Euclidean space [1, 2]. In contrast to non-spatial models, SCR models use the detection histories *and* the locations of marked individuals. An important component of SCR models is the spatial point process that specifies the location of each individual’s center of activity (or home range in some cases). The activity center of an individual is not directly observable, but it is assumed to be fixed during the period of sampling. This latter assumption can be regarded as a form of demographic closure for SCR models.

Some of the earliest SCR models were developed for the analysis of detections of *naturally marked* animals at a set of fixed locations [3, 4]. Natural markings allow individuals to be identified uniquely and come in a variety of forms owing to technological advances in non-invasive sampling methods. For example, body markings (stripe or spot patterns) can be used to distinguish individual tigers or leopards, and acoustic profiles can be used to distinguish the vocalizations of individual songbirds or whales (at least for some species). Alternatively, DNA obtained from scats or hair snares can be used to identify individuals.

Different types of natural markings obviously require different types of detectors. Here we focus on SCR surveys that satisfy two design requirements: (1) individuals can potentially be observed at more than one of several fixed locations during the period of sampling and (2) the date and time of each observation is recorded, as is common when using microphones or motion-activated cameras as detectors [5, 6]. To satisfy the first requirement, detectors should be placed at locations based on the likely range of an individual’s movements. These placements induce heterogeneity in the rates of detection among individuals because animals whose activity centers lie within the interior of a spatial array of detectors are more likely to be observed than animals whose activity centers lie near the edge of the array.

Although several SCR models have been developed with the first design requirement in mind, most these models have ignored the continuous period of operation of the detectors. In some models the total period of sampling is partitioned into discrete intervals and a binary observation of 0 (no detections of an individual) or 1 (one or more detections of an individual) is used for each interval ([4] and [2], chapter 5). This discretizaton of time is subjective and potentially uses data inefficiently because the same individual can be detected multiple times during a single interval. Discretization into 24-hour intervals does not solve the problem because two detections of the same individual may be counted either once or twice depending on the choice of cut-off time (the so-called “midnight problem” [7]). Other SCR models use the total number of detections of an individual by treating the total period of sampling as a single interval ([3] and [2], chapter 9); however, these models do not use information associated with the exact dates and times of detection.

To our knowledge, only one SCR model has been formulated for times of detection [6], and this model was developed primarily for the estimation of population density. Here, we propose a hierarchical Bayesian model which includes many of the assumptions made by [6] but also allows inferences to be computed for the spatial distribution of individual activity centers. Our approach may be used to construct species distribution models. It is useful even when the sources of spatial variation in population density are either unobserved or unknown.

We begin by describing the hierarchical model, which includes a spatial point process for the distribution of individual activity centers and a temporal point process for the distribution of detection times of each individual. We show that this model is a logical extension of count-based SCR models that do not account for temporal heterogenity in detection rates. We illustrate the benefits of our approach by analyzing the detection times of individual tigers observed in a spatial array of camera traps.

## Materials and methods

### Model description

Assume a population of *N* individually identifiable animals living within a finite region *B* of Euclidean space (specifically, $B\subset R^{2}$) is surveyed using *K* camera traps whose locations are selected to be representative of *B*. The trap locations need not be random but their spacing should permit any animal to be detected by more than one camera as a consequence of its movements. We assume that each animal living within *B* moves around a fixed, center of activity, which we denote using ***s***. Our goal is to infer the spatial distribution and abundance of the activity centers of *all* animals living within *B* using only the detection locations and times of animals observed during the camera-trap survey.

#### The data

To state the inference problem more concretely, we first describe the kinds of data observed in the camera-trap surveys. Suppose each of *K* distinct cameras is used to detect animals in region *B* during a period of time when the activity center of each animal can be assumed to be fixed. This assumption implies a spatial form of demographic closure for the population of animals living within region *B*. Ideally, all cameras operate during the same period; however, the time required to deploy all *K* cameras usually induces some differences among their actual periods of operation. Therefore, let the time interval (0, *T*_*k*_] denote the continuous period of operation of the *k*th camera, and let ***x***_*k*_ ∈ *B* denote the fixed location of this camera (*k* = 1, …, *K*).

Each camera is equipped with a motion sensor so that a photograph (detection) is only obtained during an encounter with one or more animals. The time and date of the encounter are normally recorded with each photograph; therefore, it is possible to observe a sequence of detection times for each marked animal and trap. Let $t_{ik}={\left(t_{ik,1},\ldots ,t_{ik,y_{ik}}\right)}^{\prime }$ denote the observed sequence of detection times of the *i*th individual at trap *k*, where *t*_*ikj*_ ∈ (0, *T*_*k*_] if *y*_*ik*_ > 0; otherwise, ***t***_*ik*_ = ∅. The subscript *i* ∈ {1, …, *n*} indexes the *i*th of *n* distinct animals detected at least once during the camera-trap survey. (The prime symbol indicates the transpose of a matrix or vector.)

**Fig 1:**
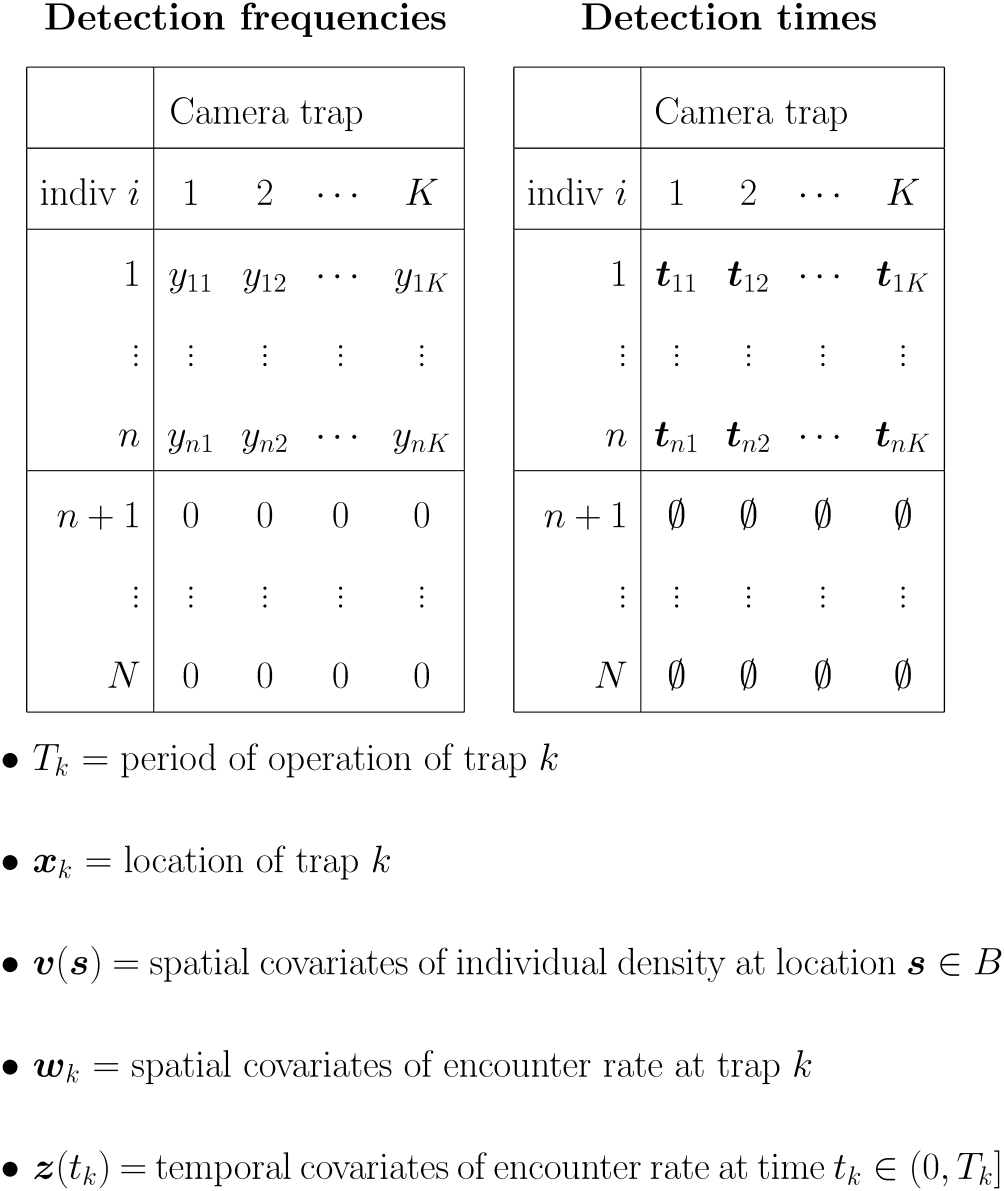
Notation used for the data observed during a camera-trap survey.

The observed data can be summarized using two arrays: one is a *n* × *K* matrix that contains the observed number of detections *y*_*ik*_ of the *i*th individual at trap *k*; the other is a *n* × *K* matrix that contains the observed sequence of detection times ***t***_*ik*_ for the *i*th individual at trap *k*. All that is known of the *N* − *n* unobserved animals is that each individual’s detection frequency is zero (Fig 1).

If spatially varying covariates are thought to influence the density of individual activity centers over *B*, we assume that a (possibly vector-valued) set of covariate measurements ***v***(***s***) is available for every location ***s*** in region *B*. In the absence of such covariates, a model of constant density can be specified using ***v***(***s***) = 1 as a regressor (see below). Similarly, if temporally varying covariates are thought to influence the temporal pattern of detection times recorded at the *k*th camera, we assume that a (possibly vector-valued) set of covariate measurements ***z***(*t*_*k*_) is available for every time *t*_*k*_ ∈ (0, *T*_*k*_] that the *k*th camera is operational.

In some surveys one or more covariates thought to be informative of an individual’s baseline rate of detection is available for each of the *K* cameras. Let ***w***_*k*_ denote the (possibly vector-valued) set of covariate measurements associated with the camera located at ***x***_*k*_. These covariates are location dependent and can be interpreted as predictors of the baseline rate at which individuals encounter the *k*th camera during the time interval (0, *T*_*k*_]. In the absence of such covariates, the baseline rate of encounter can be specified using ***w***_*k*_ = 1 (∀*k*) as a regressor (see below).

#### Spatial point-process model of individual activity centers

Following previous spatial capture-recapture models [8–10], we assume that the spatial distribution of activity centers is a realization of a Poisson process parameterized by first-order intensity function *λ*(***s***), which specifies the limiting, *expected* density of activity centers (number per unit area) at location ***s*** ∈ *B* [11]. Thus, the total number *N*(*B*) of individuals in region *B* is a Poisson random variable that depends on the intensity of the process integrated over *B*: Λ(*B*) = *∫*_*B*_ *λ*(***s***) *d****s***. We model the expected density *λ*(***s***) as a log-linear function of unknown parameters ***β*** and location-specific regressors ***v***(***s***) as follows: log[*λ*(***s***)] = ***β***′***v***(***s***).

Let *N* denote the unknown total number of individuals living in region *B*. We have established that

$$\mathrm{Pr}\left(N\left(B\right)=N\right)=\text{exp}\left[-\Lambda \left(B\right)\right]{\left[\Lambda \left(B\right)\right]}^{N}/N!$$ (1)

Furthermore, conditional on *N*, the joint probability density of the *N* individual activity centers is

$$\left[s_{1},s_{2},\ldots ,s_{N}|N\right]=N!\prod_{i=1}^{N}\lambda (s_{i})/\Lambda \left(B\right)$$ (2)

[12, 13]. (Note that we use bracket notation [14] to specify probability density functions; thus, [*x*, *y*] denotes the joint density of random variables *X* and *Y*, [*x*|*y*] denotes the conditional density of *X* given *Y* = *y*, and [*x*] denotes the unconditional (marginal) density of *X*.) The goal of our analysis is to estimate *N* and ***s***_*i*_ (*i* = 1, …, *N*) using only the observed data—that is, using the detection times of the *n* ≤ *N* individuals photographed by the cameras and their corresponding capture locations.

#### Temporal point-process model of detection times

In this section we describe a model of the observed data (i.e., the trap-specific detection times of each individual) that depends on the latent activity center of each individual. As with most spatially explicit models of camera-trap surveys, we assume that pairwise distances between each individual’s activity center and the set of camera locations induces heterogeneity in detection rates among the animals living in *B*. For example, animals whose activity centers are located in the interior of the trapping array are more likely to be detected than animals whose activity centers are located outside the trapping array. However, our model of detection times includes additional components that can influence the detection rates of individual animals. To be specific, we assume that the sequence of detection times observed for the *i*th individual at camera trap *k* is a realization of a Poisson process parameterized by first-order, intensity function

$$\phi \left(t,s_{i},x_{k}\right)=\psi_{k}\;\gamma \left(t\right)\;\text{exp}(-||s_{i}-x_{k}{\left|\right|}^{2}/\left(2\sigma^{2}\right))$$ (3)

for *t* ∈ (0, *T*_*k*_], where ∥***s***_*i*_ − ***x***_*k*_∥ specifies the Euclidean distance between locations ***s***_*i*_ and ***x***_*k*_ and *σ* (> 0) is an unknown parameter whose value increases with the spatial extent an animal’s movements about its activity center. To specify the baseline rate *ψ*_*k*_ (> 0) at which individuals encounter the *k*th camera trap during time interval (0, *T*_*k*_], we use a log-linear model log(*ψ*_*k*_) = ***α***′***w***_*k*_, which includes a vector of unknown parameters ***α*** and an observed set of covariate measurements ***w***_*k*_ associated with the *k*th trap. In addition, the function *γ*(*t*) may be used to specify sources of time-dependence in the sequence of each animal’s detection times. For example, if animals are primarily active at night (as with tigers in India), one would expect encounters of animals with traps to be higher during the night than during periods of daylight. In this case a time-dependent indicator of daylight, say *z*(*t*), might be used to model *γ*(*t*) in terms of an unknown parameter *ξ* as follows: log(*γ*(*t*)) = *ξ* *z*(*t*). Of course, this approach may be generalized to specify the effects ***ξ*** of several temporal covariates ***z***(*t*). An important caveat is that the value of each temporal covariate must be available for the entire period of each trap’s operation.

We have not yet described the relationship between the observed detection times of an individual and the Poisson intensity given in Eq (3). Conditional on activity center ***s***_*i*_, the number of detections *Y*_*ik*_(*T*_*k*_) of the *i*th individual at trap *k* during time interval (0, *T*_*k*_] has a Poisson distribution as follows:

$$Y_{ik}\left(T_{k}\right)\;|\;s_{i}∼\text{Poisson}\left(\Phi \left(T_{k},s_{i},x_{k}\right)\right)$$

where $\Phi \left(T_{k},s_{i},x_{k}\right)=\int_{0}^{T_{k}}\phi \left(t,s_{i},x_{k}\right)\;dt$ is the expected number of detections of the *i*th individual at trap *k* during its period of operation. Furthermore, conditional on the observed number of detections *y*_*ik*_ and ***s***_*i*_, the joint probability density of the observed detection times is

$$[t_{ik}\left|y_{ik},s_{i}\right]=\prod_{j=1}^{y_{ik}}\phi \left(t_{ikj},s_{i},x_{k}\right)/\Phi \left(T_{k},s_{i},x_{k}\right)$$

Note that unlike the spatial Poisson process, there is only one possible ordering of the observed detection times in ***t***_*ik*_ (i.e., sequential order).

#### Likelihood functions for fitting models to data

We have described all of the components needed to derive the likelihood function of the model. To clarify the derivation of this function, for the moment suppose (counterfactually) that the activity centers of all *N* individuals living in region *B* were observable and that we wanted only to estimate the unknown parameters ***θ*** = (***β***′, ***α***′, *σ*, ***ξ***′)′. In this case the likelihood function for ***θ*** is

$$\left[y_{\left(1:N\right)},t_{\left(1:N\right)},N,s_{1},\ldots ,s_{N}|\theta \right]=\left[N\right]\left[s_{1},s_{2},\ldots ,s_{N}|N\right]\prod_{i=1}^{N}\prod_{k=1}^{K}\left[y_{ik},t_{ik}|s_{i}\right]$$ (4)

where ***y***_(1:*N*)_ and ***t***_(1:*N*)_ are *N* × *K* matrices with elements *y*_*ik*_ and ***t***_*ik*_, respectively. The joint density of *y*_*ik*_ and ***t***_*ik*_ (conditional on ***s***_*i*_) is [*y*_*ik*_, ***t***_*ik*_|***s***_*i*_] = [*y*_*ik*_|***s***_*i*_][***t***_*ik*_|*y*_*ik*_, ***s***_*i*_].

Of course, in reality only *n* of the *N* individuals living in region *B* are detected in the array of camera traps, and none of the individual activity centers is observable. All that is known about the *n*_0_ = *N* − *n* unobserved individuals is that each animal was not detected at any of the *K* traps, that is *y*_*ik*_ = 0 (∀*k*, *i* ∈ {*n* + 1, …, *N*}). The likelihood function of the observed data requires the following modification of Eq (4) to account for the (Nn) ways of observing *n* individuals in the camera-trap surveys:

$$\begin{array}{ll} \left[y_{\left(1:n\right)},t_{\left(1:n\right)},n,n_{0},s_{1},\ldots ,s_{n}|\theta \right] & =\left[N\right]\left(\begin{array}{c} N\\ n \end{array}\right)\pi_{0}^{n_{0}}[s_{1},s_{2},\ldots ,s_{n}|N]\prod_{i=1}^{n}\prod_{k=1}^{K}[y_{ik},t_{ik}|s_{i}]\\ & =\frac{\text{exp}\left[-\Lambda \left(B\right)\right]{\left[\pi_{0}\Lambda \left(B\right)\right]}^{n_{0}}}{n_{0}!}\prod_{i=1}^{n}\lambda \left(s_{i}\right)\prod_{k=1}^{K}[y_{ik},t_{ik}|s_{i}] \end{array}$$ (5)

where ***y***_(1:*n*)_ and ***t***_(1:*n*)_ are the *n* × *K* matrices of observed data and where

$$\left[y_{ik},t_{ik}|s_{i}\right]=\frac{1}{y_{ik}!}\text{exp}\left[-\Phi \left(T_{k},s_{i},x_{k}\right)\right]\;\prod_{j=1}^{y_{ik}}\phi \left(t_{ikj},s_{i},x_{k}\right)$$

The probability that an individual living in region *B* was not detected in any of the *K* camera traps during the survey period is

$$\begin{array}{ll} \pi_{0} & =\int_{B}[s|N]\prod_{k=1}^{K}[y_{k}=0|s]\text{d}s\\ & =\frac{1}{\Lambda \left(B\right)}\int_{B}\lambda \left(s\right)\prod_{k=1}^{K}\text{exp}\left[-\Phi \left(T_{k},s,x_{k}\right)\right]\text{d}s \end{array}$$

Note that the likelihood function in Eq (5) includes some quantities that are not observed (*n*_0_ and ***s***_*i*_). If desired, a marginal likelihood function for ***θ*** can be computed by integrating *n*_0_ and ***s***_*i*_ from Eq (5) (see Dorazio 2013 [10] for example). However, for our purposes this calculation is counterproductive because we want to estimate the activity centers of both observed and unobserved individuals living in *B*. We therefore adopt the Bayesian approach to inference and fit the model using the following unnormalized posterior density function:

$$\left[\theta ,n_{0},s_{1},\ldots ,s_{n}|y_{\left(1:n\right)},t_{\left(1:n\right)},n\right]\propto \left[\theta \right]\left[y_{\left(1:n\right)},t_{\left(1:n\right)},n,n_{0},s_{1},\ldots ,s_{n}|\theta \right]$$ (6)

where [***θ***] denotes the prior density of ***θ***.

#### Restricted models for a homogenous temporal point process

In the absence of time dependence in the detection process (i.e., *γ*(*t*) = *γ*, ∀*t*), the spatial distribution of individual activity centers can be estimated without using the detection times ***t***_(1:*n*)_ of the *n* observed individuals. This is possible because the temporal point process is homogeneous and the intensity function in Eq (3) simplifies to

$$\phi \left(s_{i},x_{k}\right)=\psi_{k}\;\gamma \;\text{exp}(-||s_{i}-x_{k}{\left|\right|}^{2}/\left(2\sigma^{2}\right))$$

which is not a function of time *t*.

In this case the expected number of detections of the *i*th individual at trap *k* is proportional to the duration *T*_*k*_ of that trap’s operation, that is, Φ(*T*_*k*_, ***s***_*i*_, ***x***_*k*_) = *T*_*k*_ *ϕ*(***s***_*i*_, ***x***_*k*_). Furthermore, it is easily shown that the conditional distribution of observed detection times is Uniform(0, *T*_*k*_). This result implies a considerable simplification of the likelihood function because the joint density of *y*_*ik*_ and ***t***_*ik*_ (conditional on ***s***_*i*_) does not depend on the observed sequence ***t***_*ik*_ of detection times:

$$\begin{array}{ll} \left[y_{ik},t_{ik}|s_{i}\right] & =[y_{ik}|s_{i}][t_{ik}|y_{ik},s_{i}]\\ & =\frac{1}{y_{ik}!}\text{exp}\left[-T_{k}\phi \left(s_{i},x_{k}\right)\right]{\left[\phi \left(s_{i},x_{k}\right)\right]}^{y_{ik}} \end{array}$$

Note that the parameters used to specify *ψ*_*k*_ and *γ* (i.e., ***α*** and *ξ*, respectively) are no longer identifiable; thus, we can ignore *γ*—recognizing that it cannot be identifed from the parameter *α*_0_—and formulate a likelihood function [***y***_(1:*n*)_, *n*, *n*_0_, ***s***_1_, …, ***s***_*n*_|***θ***] of the observed matrix of counts ***y***_(1:*n*)_ to estimate ***θ*** = (***β***′, ***α***′, *σ*)′. As before, we adopt the Bayesian approach to inference and fit this restricted class of models using the following unnormalized posterior density function:

$$\left[\theta ,n_{0},s_{1},\ldots ,s_{n}|y_{\left(1:n\right)},n\right]\propto \left[\theta \right]\left[y_{\left(1:n\right)},n,n_{0},s_{1},\ldots ,s_{n}|\theta \right]$$ (7)

#### Posterior inference and predicting activity centers of unobserved individuals

We used Markov chain Monte Carlo (MCMC) methods [15] to estimate the parameters of models with posterior density functions given in Eqs (6) and (7) and to estimate maps of the spatial distribution of activity centers. Once a model was fitted, we estimated the activity centers of the *N* − *n* unobserved individuals by simulating draws from a common distribution whose conditional density function is

$$\begin{array}{ll} \left[s|y_{1}=y_{2}=\cdots =y_{K}=0\right] & =\frac{\left[s|N]\prod_{k=1}^{K}[y_{k}=0|s\right]}{\pi_{0}}\\ & =\frac{\lambda \left(s\right)\prod_{k=1}^{K}\text{exp}\left[-\Phi \left(T_{k},s,x_{k}\right)\right]}{\pi_{0}\Lambda \left(B\right)} \end{array}$$

By simulating draws of ***s*** for each element of the Markov chain, we averaged over the posterior uncertainty of the model’s parameters to compute summaries (including maps) of the posterior predictions of these activity centers. For example, we estimated the spatial distribution of abundance of individuals by partitioning the spatial domain *B* into a set of disjoint (nonoverlapping) grid cells (*B*_*m*_ for the *m*th cell) and by computing the number of individual activity centers in each grid cell as follows: $N\left(B_{m}\right)=\sum_{i=1}^{N}I\left(s_{i}\in B_{m}\right)$, where *I*(*ε*) denotes an indicator function that equals one if expression *ε* is true and equals zero otherwise. Abundance *N*(*B*_*m*_) was computed for each element of the Markov chain so that posterior uncertainty of all model parameters was accounted for while estimating each cell’s abundance. Details about our MCMC algorithm and prior distributions are described in S1 Appendix.

### Camera-trap survey of tigers

**Fig 2:**
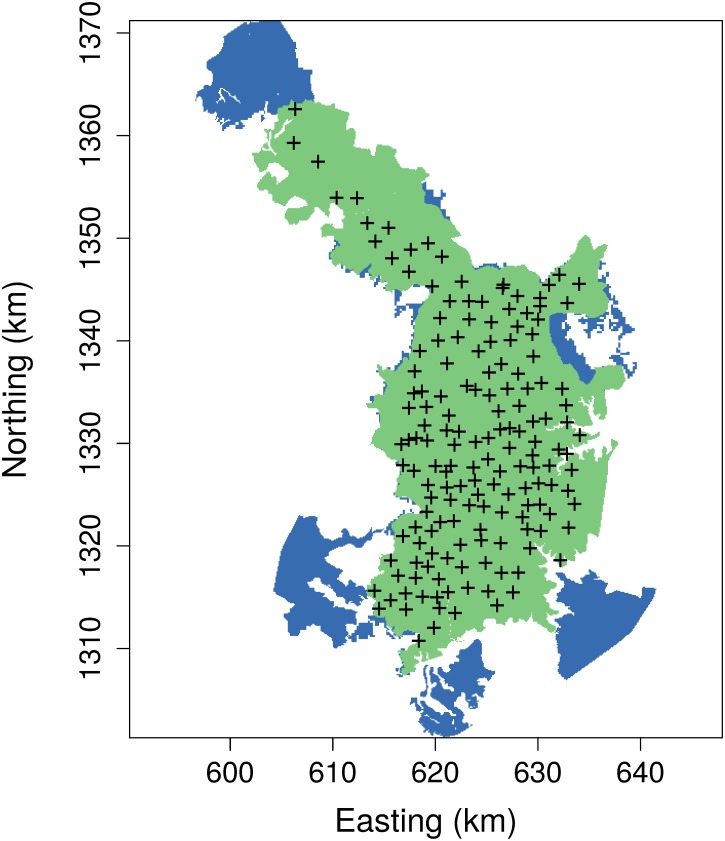
Map of Nagarahole Tiger Reserve and adjacent areas in the Western Ghat region of India (shown in green). Locations of camera traps are indicated by plus signs. The buffer region outside the study area (shown in blue) also contains suitable tiger habitat.

**Fig 3:**
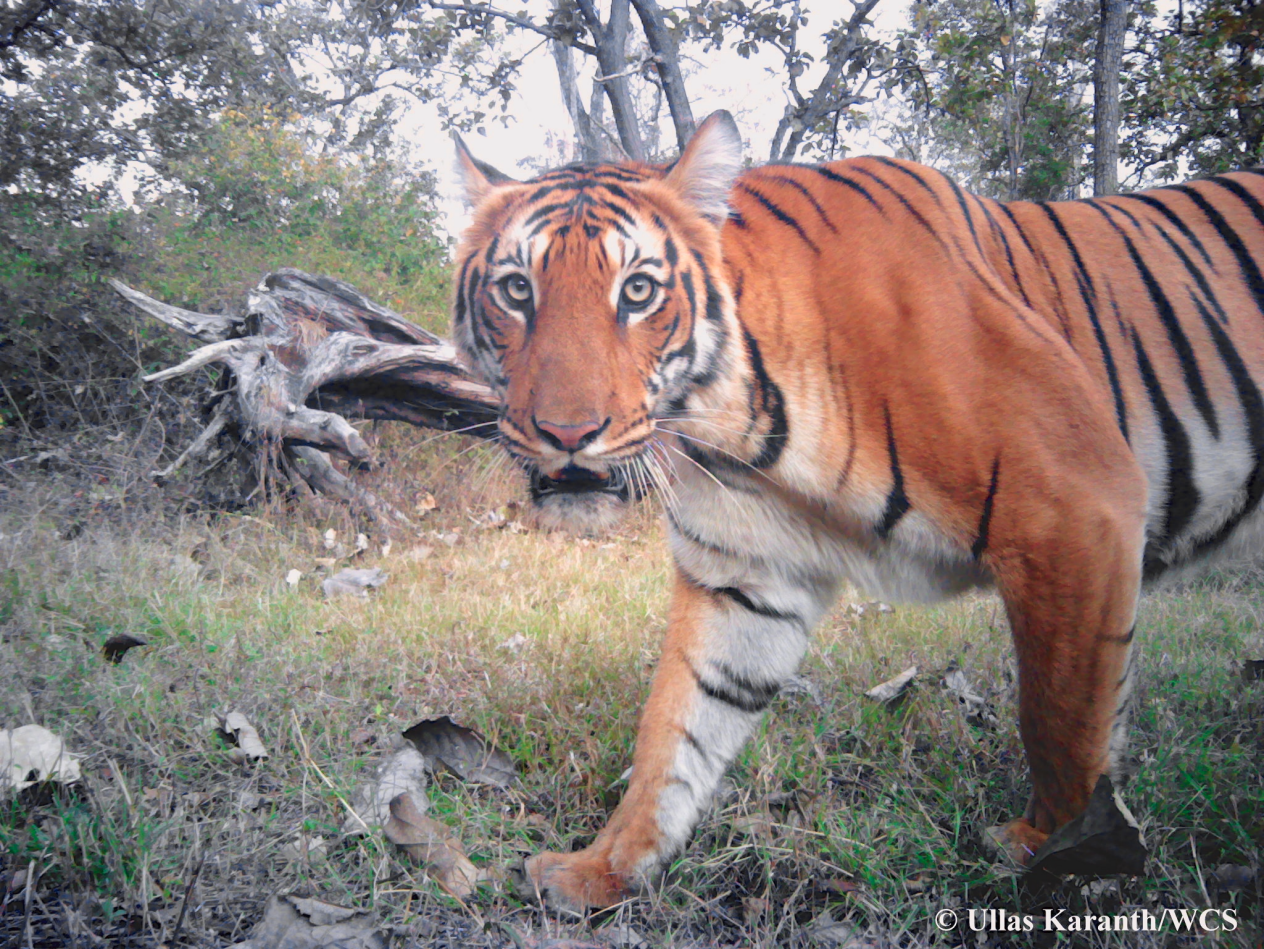
Photograph of tiger detected at a camera trap within the Nagarahole Tiger Reserve.

We surveyed the population of tigers (*Panthera tigris*) living within and adjacent to the Nagarahole Tiger Reserve of Karnataka, India using a spatial array of 162 motion-activated camera traps (Fig 2). Ecological descriptions of the study area and species were provided by [16, 17] and [18]. Each camera trap included two inward facing cameras so that both flanks of an individual tiger could be photographed [19]. Camera trap locations were selected to increase detections using prior knowledge of the behavior and range of movements of individual tigers [4, 17]. Each camera trap operated continuously during the 45-day period of sampling (26 Nov 2014 to 13 Jan 2015), recording the date and time when a tiger was photographed. Individual tigers were identified from photographs using their unique stripe patterns [16], as shown in Fig 3. All field work was carried out under research permits issued by the Karnataka Forest Department and the Kerala Forest Department, in forest areas administered by these agencies. No other approvals or permits were required because our non-invasive, camera-trap surveys did not involve any handling, collection, sampling or sacrifice of animals, or experimental manipulation of any type.

Camera traps were placed 2–3 km apart throughout the 860 km^2^ area to provide adequate exposure to all tigers. The spatial domain used to fit our SCR models also included a 10-km wide buffer where suitable habitat existed, thus yielding a total area of 1130 km^2^ (Fig 2). The width of this buffer was based on previous radio-telemetry and camera-trap studies of tiger movements and home ranges [17, 18]. Areas outside of this domain contained land-use formations unlikely to be occupied by resident tigers.

We fitted two SCR models to the detection times of individual tigers observed in the spatial array of cameras. One model used a time-dependent binary indicator *z*(*t*) to specify whether each detection occurred during the daytime (i.e., between sunrise and sunset (*z*(*t*) = 1)) or during the nighttime (*z*(*t*) = 0). We obtained sunrise and sunset times for each day of sampling from an online application http://aa.usno.navy.mil/data/docs/RS_OneYear.php developed by the Astronomical Applications Department of the United States Naval Observatory. We also fitted a restricted model wherein detection rate was assumed to be constant at each camera location, as described in Model description. This model did not require the detection times and was fitted using only the matrix of observed detection frequencies. Neither of these SCR models included spatial covariates of tiger density or trap-specific covariates of tiger detection rate; however, we computed estimates of the spatial distribution of individual activity centers and tiger abundances using the full (unrestricted) model and a 500 m × 500 m grid of the spatial domain. The R source code [20] and data needed to fit these models are available in S2 Appendix.

### Simulation study

**Fig 4:**
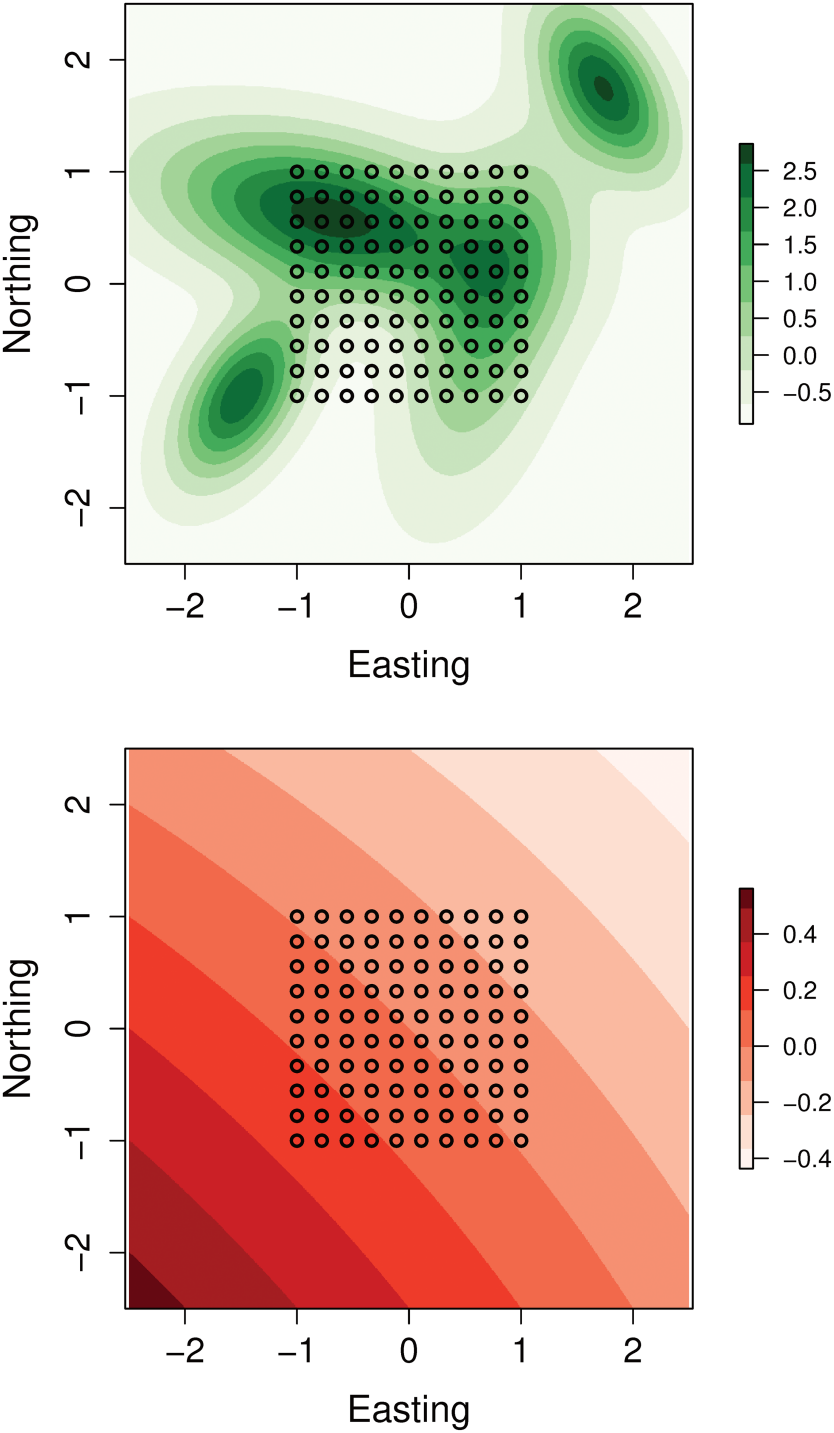
Maps of covariates used in simulation study. Upper panel = covariate of individual density. Lower panel = covariate of baseline detection rate. Trap locations are indicated by circles.

We conducted a simulation study to examine the performance of our model when analyzing data sets of greater complexity than the tiger data. In this study we simulated values of one spatially varying covariate *v*(***s***) of individual density and one spatially varying covariate *w*(**s**) of baseline detection rate over a square region *B* (Fig 4). S3 Appendix contains a description of how values of each covariate were computed as a function of location **s**. The expected density of individuals at location ***s*** was computed using *λ*(***s***) = exp(1.4 + 0.8*v*(***s***)). Given these parameter values, the expected number of individuals in region *B* was 150.2 (= Λ(*B*)).

We assumed that individuals living in *B* may have been detected within a square array of 100 evenly-spaced traps located at the center of the region (Fig 4). The baseline detection rate of individuals at trap location ***x***_*k*_ was computed using *ψ*_*k*_ = exp(−0.7 + 1.0*w*(***x***_*k*_)). Given these parameter values, baseline detection rates ranged from 0.41 to 0.61 detections per unit time among the *K* = 100 trap locations. We assumed each trap operated continuously for 30 units of time. We also assumed equal amounts of daytime and nighttime during this period and that each trap’s baseline detection rate during the day was about 37% of the rate at night. In other words, we assumed *ξ* = −1. We assumed the parameter *σ* was 0.4 to ensure that individuals in proximity to the trapping array were likely to be detected at multiple traps.

We fitted the full model and the restricted model to each of 300 data sets that were simulated independently. Each model was fitted to a data set by running the MCMC algorithm (S1 Appendix) for 1200 iterations and by estimating posterior summaries from the final 1000 iterations of the Markov chain. We examined the performance of these models by comparing the average of the estimated posterior mean of each parameter to its true (data-generating) value. We also compared the estimated coverage of a 95% credible interval for each parameter to the nominal 95% level.

## Results

### Camera-trap survey of tigers

**Fig 5:**
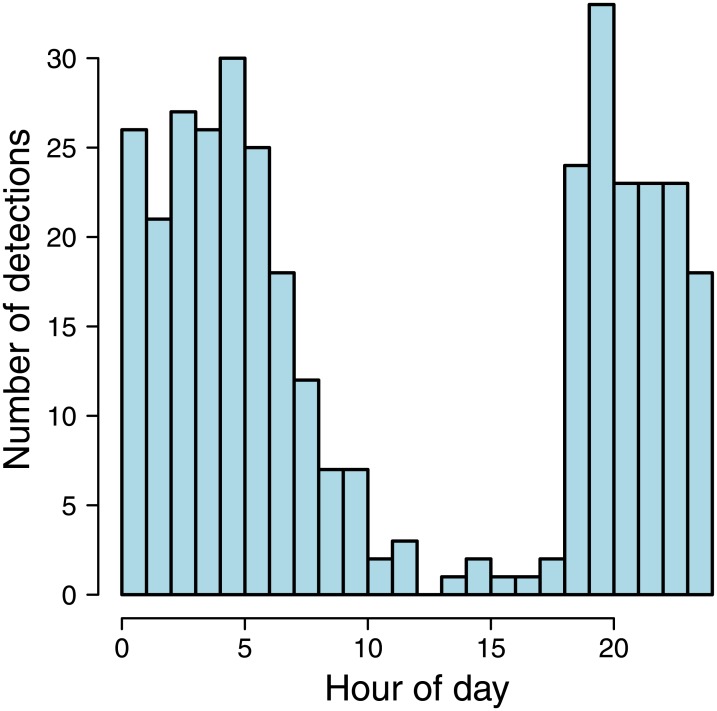
Temporal distribution of observed detection times for photo-captured tigers in a 24-hour period.

A total of 86 individual tigers were detected during the period of sampling, which included a per-camera average of 21.2 days of daytime and 23.4 days of nighttime. Tigers were detected in a total of 355 capture events. Most of these detections occurred during the nighttime (Fig 5), providing evidence of nocturnal activity patterns of tigers.

Table 1 contains estimates of the parameters of both full and restricted SCR models. Substantial differences in parameter estimates were obtained only among the parameters that specify encounter rates (*α*_0_ and *ξ*). In particular the estimate of *ξ* was significantly negative, indicating that baseline detection rates of tigers were about five times lower during the daytime (0.009 detections per day) than during the night (0.045 detections per day).

**Table 1:** Estimates of posterior summary statistics for the parameters of the SCR models fitted to the tiger data.

	Full model			Restricted model		
	Mean	2.5%	97.5%	Mean	2.5%	97.5%
*σ*	1.70 (0.0023)	1.60 (0.0033)	1.82 (0.0037)	1.70 (0.0024)	1.59 (0.0031)	1.82 (0.0038)
*ξ*	-1.68 (0.0033)	-1.98 (0.0099)	-1.39 (0.0060)			
*α*_0_	-3.10 (0.0033)	-3.28 (0.0059)	-2.94 (0.0051)	-3.58 (0.0027)	-3.74 (0.0054)	-3.41 (0.0048)
*β*_0_	-2.19 (0.0033)	-2.40 (0.0071)	-1.98 (0.0057)	-2.19 (0.0036)	-2.41 (0.0079)	-1.99 (0.0064)
*n*_0_	41 (0.24)	26 (0.36)	57 (0.53)	41 (0.26)	27 (0.36)	57 (0.45)

Limits of 95% credible intervals are indicated by the columns labeled 2.5% and 97.5%. Monte Carlo standard errors are given in parentheses.

**Fig 6:**
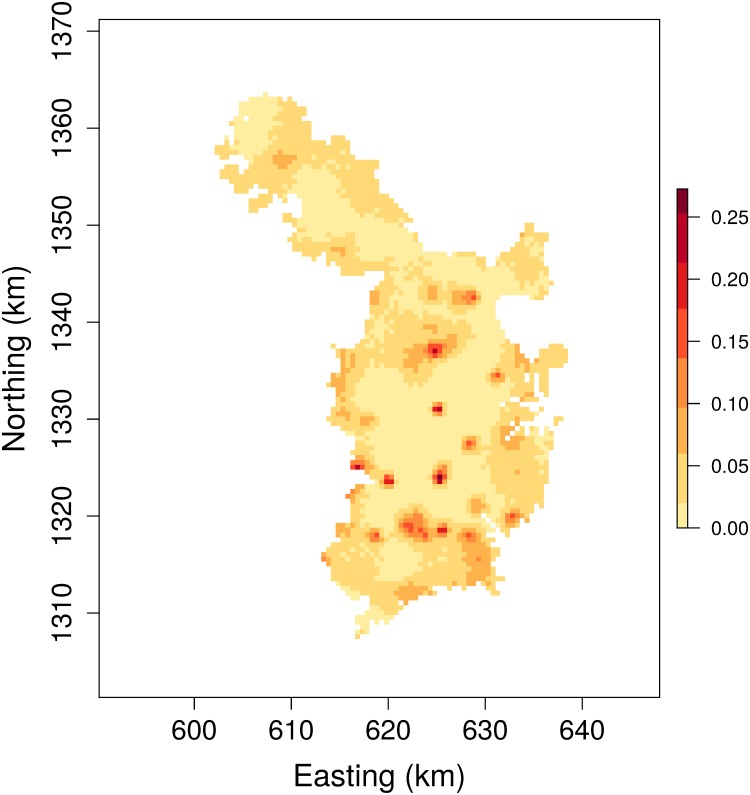
Map of estimated distribution of tiger abundance in Nagarahole Tiger Reserve and adjacent areas in the Western Ghat region of India.

The expected density of tigers throughout our spatial domain was 11.3 individuals per 100 km^2^ (95% credible interval = 9.1–13.9). Although spatially varying covariates of tiger density were not used to fit our SCR model, the estimated spatial distribution of tiger abundance (Fig 6) revealed some locations where tiger densities were much higher than average.

The estimate of the scale parameter *σ* was 1.70 km (95% credible interval = 1.60–1.82). Using a Gausian kernel, we estimate that each tiger occupied an area of about 54 km^2^ (= $\pi {\hat{\sigma }}^{2}\chi_{2,0.95}^{2}$), which slightly exceeds the home range expected for resident male tigers (50 km^2^) [18].

### Simulation study

In our simulated data sets the average number of individuals living in region *B* (150.3) was very close to the expected population size (150.2). The average number of individuals detected at least once in the trapping array was 124.0. Table 2 contains averages of each parameter’s posterior mean and coverage estimated by fitting the full and the restricted SCR models to each simulated data set. The average of the estimated posterior mean was close to the true (data-generating) value of every parameter with one exception. The average of the logit-scale, mean baseline encounter rate *α*_*o*_ estimated under the restricted model was substantially lower than the true value of this parameter. Similarly, the coverage of credible intervals of *α*_0_ obtained by fitting the restricted model was substantially lower than the nominal 95% level. The reason for these discrepancies are obvious: under the assumptions of the data-generating model, mean baseline encounter rates differed during daytime and nighttime, whereas under the assumptions of the restricted model these rates were assumed to be identical. Therefore, under the restricted model estimates of *α*_0_ correspond to the logit-scale, mean baseline encounter rate averaged over daytime and nighttime periods. Apart from this difference, the operating characteristics of full and restricted models were nearly identical.

**Table 2:** Results of simulation study.

Parameter	True value	Full model		Restricted model	
		Average	Coverage	Average	Coverage
*β*_0_	1.4	1.37 (0.0087)	0.95 (0.0130)	1.37 (0.0087)	0.94 (0.0133)
*β*_1_	0.8	0.81 (0.0057)	0.91 (0.0168)	0.81 (0.0057)	0.91 (0.0168)
*α*_0_	-0.7	-0.70 (0.0008)	0.94 (0.0141)	-1.08 (0.0008)	0.00 (0.0000)
*α*_1_	1.0	0.98 (0.0101)	0.95 (0.0126)	0.98 (0.0101)	0.95 (0.0130)
*ξ*	-1.0	-1.00 (0.0012)	0.95 (0.0122)		
*σ*	0.4	0.40 (0.0001)	0.97 (0.0093)	0.40 (0.0001)	0.97 (0.0093)

Average = average of estimates of posterior mean. Coverage = proportion of estimated 95% credible intervals that include the true parameter value. Monte Carlo standard errors are given in parentheses.

## Discussion

In this article we described a class of hierarchical models for estimating the spatial distribution and abundance of animals detected with continuous-time recorders. In these models the spatial distribution of individual activity centers is assumed to be a realization of a spatial point process parameterized to include the effects of spatially varying covariates (e.g., habitat measurements). Similarly, the sequence of detection times observed for each unique individual is assumed to follow a temporal point process during the continuous period of each camera’s operation. This process is parameterized to include the effects of trap-specific covariates on each individual’s baseline rate of detection so that factors influencing animal movements or behaviors can potentially be inferred. The effects of behavior also can be specified using time-specific covariates, provided measurements of these covariates are available during the entire period of each trap’s operation. These novel aspects of our continuous-time SCR model provide a more realistic description of the nocturnal activities and movements of tigers and other species with similar habits.

Several components of our SCR model are also present in other SCR models. For example, the effects of spatially varying covariates on density of activity centers has been modeled in some SCR models using a multinomial distribution to specify a discrete representation of the locations expected to be occupied by each individual ([2, 21], chapter 11). A similar approach was used to specify each individual’s utilization of space with a multinomial resource selection function ([2, 22], chapter 13). This allowed [22] to establish a technical equivalence between an individual’s resource selection and encounter probability. Specifically, they showed that specifying the baseline rate of detection of an individual as a function of trap- and location-specific covariates (as in our SCR model) implies a particular resource selection function. Therefore, if resource selection occurs it can be estimated from the effects of trap- and location-specific covariates on each individual’s baseline rate of detection.

Our SCR model also shares many of the features of the continuous-time SCR model of [6]. For example, in that model the distribution of detection times was parameterized using a hazard function *h*_*k*_(*t*, ***x***_*i*_; ***θ***), wherein ***x***_*i*_ denotes the activity center of individual *i* and *k* indexes the trap. Similarly, our continuous-time SCR model uses the intensity function *ϕ*(*t*, ***s***_*i*_, ***x***_*k*_) (defined in Eq (3)) of an inhomogenous Poisson process to specify the distribution of detection times for an individual with activity center ***s***_*i*_. Both models allow an individual’s baseline rate of detection to be modeled using trap-specific covariates (specified using log-linear formulations of $g_{0,T_{s}}$ [6] or of *ψ*_*k*_ (our SCR model)). The primary difference between our continuous-time SCR model and that of [6] lies in the treatment of individual activity centers. The model of [6] was used primarily to estimate model parameters, in particular the parameters that specify population density. Parameters were estimated by maximizing the marginal likelihood function obtained by integrating individual activity centers from the joint probability density of the data and the activity centers. In contrast, our SCR model was fitted using a Bayesian approach that retains the individual activity centers as formal parameters; therefore, our model can be used to estimate the spatial distribution of individual activity centers, including valid estimates of their uncertainties. As such, our SCR model essentially provides a species distribution model that allows a map of the distribution of abundance (or density) of individual activity centers to be estimated. Moreover, if spatial covariates of individual density are unknown or unavailable, our SCR model can still be used to reveal spatial variation in the distribution of individual activity centers, as was evident in our analysis of the tiger data.

Given the cost-effectiveness and widespread use of camera-trap surveys, we anticipate increasing use of continuous-time SCR models. Unlike SCR models based on discrete periods of observation, continuous-time models are more accurate representations of the actual sampling process when camera traps or other types of continous-time recorders are used. Continuous-time SCR models use *all* of the data, which may be advantageous in surveys of species at high density, where individuals are observed many times. An additional benefit of these models is their ability to specify aspects of the detection process more accurately when there are temporal variations in the activity of individual animals. In our analysis of the tiger data, we illustrated use of temporal covariates to model nocturnal behavior; however, semiparametric models of temporal patterns (e.g., splines or other basis functions of time) also can be used in circumstances where covariates of detection are unknown or unavailable. A challenging, but important, area of further research is the extension of continuous-time SCR models for estimating population dynamics. Such “open” models have been developed for discrete-time SCR models ([2, 23], chapter 16). Similar approaches may provide useful extensions of continuous-time SCR models. We plan to address this issue in ongoing work.

## Supporting information

S1 Appendix Markov chain Monte Carlo algorithms. (PDF)

S2 Appendix R source code used to fit models of tiger data observed during camera-trap survey. This software has been approved for release by the U.S. Geological Survey (USGS). Although the software has been subjected to rigorous review, the USGS reserves the right to update the software as needed pursuant to further analysis and review. No warranty, expressed or implied, is made by the USGS or the U.S. Government as to the functionality of the software and related material nor shall the fact of release constitute any such warranty. Furthermore, the software is released on condition that neither the USGS nor the U.S. Government shall be held liable for any damages resulting from its authorized or unauthorized use.
(ZIP)

S3 Appendix Description of spatial covariates used in simulation study. (PDF)

S4 Appendix R source code used to create simulated data and to fit models to these data. This software has been approved for release by the U.S. Geological Survey (USGS). Although the software has been subjected to rigorous review, the USGS reserves the right to update the software as needed pursuant to further analysis and review. No warranty, expressed or implied, is made by the USGS or the U.S. Government as to the functionality of the software and related material nor shall the fact of release constitute any such warranty. Furthermore, the software is released on condition that neither the USGS nor the U.S. Government shall be held liable for any damages resulting from its authorized or unauthorized use.
(ZIP)
