# Supplementary material for: A hierarchical model for estimating the spatial distribution and abundance of animals detected by continuous-time recorders
Source: PLoS One. 2017 May 17;12(5):e0176966. doi: 10.1371/journal.pone.0176966 (PMC5435310; doi:10.1371/journal.pone.0176966)
Supplement: S1 Appendix — (PDF) [file pone.0176966.s001.pdf]

S1 Appendix: Markov chain Monte Carlo (MCMC) algorithms[1]

In this supplement we describe the MCMC algorithms that were used to compute the Markov chains needed for estimating summaries of the posterior distribution of each model's parameters. We use bracket notation (Gelfand and Smith, 1990) to specify probability density functions; thus, $[x, y]$ denotes the joint density of random variables X and Y, $[x|y]$ denotes the conditional density of X given $Y = y$, and $[x]$ denotes the unconditional (marginal) density of X.

Model of detection frequencies and detection times

We used a MCMC algorithm to generate a Markov chain whose stationary distribution is equivalent to a posterior with the following unnormalized density function:

$$[\boldsymbol{\theta}, n_0, \boldsymbol{s}_1, \ldots, \boldsymbol{s}_n | \boldsymbol{y}_{(1:n)}, \boldsymbol{t}_{(1:n)}, n] \propto [\boldsymbol{\theta}][\boldsymbol{y}_{(1:n)}, \boldsymbol{t}_{(1:n)}, n, n_0, \boldsymbol{s}_1, \ldots, \boldsymbol{s}_n | \boldsymbol{\theta}]$$

where $\boldsymbol{\theta} = (\boldsymbol{\beta}', \boldsymbol{\alpha}', \sigma, \xi)'$ denotes a vector of unknown parameters assumed to have mutually independent prior distributions (that is, $[\boldsymbol{\theta}] = [\boldsymbol{\beta}][\boldsymbol{\alpha}][\sigma][\xi]$). The posterior density function conditions on n, the number of distinct individuals observed during the sampling period, and on the frequencies and times of detection ($\boldsymbol{y}_{(1:n)}$ and $\boldsymbol{t}_{(1:n)}$, respectively) of these individuals.

We developed a MCMC algorithm that combined two sampling algorithms (delayed-rejection, Metropolis-Hastings (Tierney and Mira, 1999; Mira, 2001) and adaptive, Metropolis (Rosenthal, 2011)) to draw random samples from full conditional distributions. This approach was more complex to implement than simple Metropolis-Hastings, but it produced considerably more efficient Markov chains that mixed well and appeared to converge more

[1]This draft manuscript is distributed solely for purposes of scientific peer review. Its content is deliberative and predecisional, so it must not be disclosed or released by reviewers. Because the manuscript has not yet been approved for publication by the U.S. Geological Survey (USGS), it does not represent any official USGS finding or policy.

quickly. Each of the following full conditional distributions was sampled in one iteration of our MCMC algorithm:

1. The full conditional for n_0 has a familiar form: $n_0|\cdot \sim \text{Poisson}(\pi_0\,\Lambda(B))$, where

$$\pi_0\,\Lambda(B) = \int_B \lambda(\boldsymbol{s}) \prod_{k=1}^{K} \exp[-\Phi(T_k, \boldsymbol{s}, \boldsymbol{x}_k)]\,\mathrm{d}\boldsymbol{s}$$

(The integral required to compute $\pi_0\,\Lambda(B)$ cannot be evaluated in closed form. In practice this integral is approximated as a Riemann sum by partitioning B into a sufficiently fine grid.) The full conditional for n_0 is the conditional posterior for the number of activity centers of animals that were present in region B but not detected during the period of sampling. In our model of the tiger data, $\Phi(T_k, \boldsymbol{s}, \boldsymbol{x}_k)$ can be expressed in closed form as follows:

$$\Phi(T_k, \boldsymbol{s}, \boldsymbol{x}_k) = (T_{k,n} + T_{k,d}\exp(\xi))\ \exp(\boldsymbol{\alpha}'\boldsymbol{w}_k - ||\boldsymbol{s} - \boldsymbol{x}_k||^2/(2\sigma^2))$$

where $T_{k,n}$ and $T_{k,d}$ denote the periods of operation of camera k during nighttime and daytime, respectively and where $T_k = T_{k,n} + T_{k,d}$.

2. The full conditional for $\boldsymbol{s}_i$ has unnormalized density

$$[\boldsymbol{s}_i|\cdot] = \lambda(\boldsymbol{s}_i) \prod_{k=1}^{K} \exp(-\Phi(T_k, \boldsymbol{s}_i, \boldsymbol{x}_k)) \prod_{j=1}^{y_{ik}} \phi(t_{ikj}, \boldsymbol{s}_i, \boldsymbol{x}_k)$$

where $\phi(t_{ikj}, \boldsymbol{s}_i, \boldsymbol{x}_k) = \exp[\boldsymbol{\alpha}'\boldsymbol{w}_k + \xi\, z(t_{ikj}) - ||\boldsymbol{s}_i - \boldsymbol{x}_k||^2/(2\sigma^2)]$. To sample this full conditional, we used a delayed-rejection Metropolis-Hastings algorithm treating $[\boldsymbol{s}_i|\cdot]$ as the target density. In particular, first we used a bivariate normal distribution as a proposal and selected its parameters to approximate the target distribution. Specifically, let $f(\boldsymbol{s}_i) = \log([\boldsymbol{s}_i|\cdot])$. We assigned the mean of the proposal distribution to equal $\hat{\boldsymbol{s}}_i$, the value of $\boldsymbol{s}_i$ that maximized $f(\boldsymbol{s}_i)$. This maximimization was done numer-

ically using an analytical gradient $\boldsymbol{g}(\boldsymbol{s}_i)$ and hessian $\boldsymbol{H}(\boldsymbol{s}_i)$. The covariance matrix of the proposal distribution was computed by inverting the negative of the hessian matrix $[-\boldsymbol{H}(\hat{\boldsymbol{s}}_i)]^{-1}$. If the candidate of this proposal distribution was rejected, we computed a second candidate using a bivariate normal distribution with mean equal to the current value of $\boldsymbol{s}_i$ and with a diagonal covariance matrix $\sigma_{s_i}^2 \boldsymbol{I}$ (where $\boldsymbol{I}$ is an identity matrix and σ_{s_i} is a known scale parameter). In other words, we used a random-walk Metropolis algorithm to generate the second candidate. The acceptance probability of the second candidate was computed to ensure that the Markov chain remained reversible relative to its stationary distribution (Mira, 2001). In cases where the first proposal's mean or covariance matrix could not be computed due to failed optimization, we simply applied the random-walk Metropolis algorithm with the bivariate normal proposal described earlier. The scale parameter σ_{s_i} of the random-walk proposal distribution was tuned adaptively – that is, by incrementing or decrementing the proposal distribution's variance depending on whether or not the acceptance rate in each batch of 50 iterations of the MCMC algorithm exceeded a target rate of 0.234 (Rosenthal, 2011, Sections 4.3.3 and 4.3.4). We reduced the absolute value of these adjustments in proportion to the inverse square root of the number of batches to ensure that the diminishing-adaptation condition required for convergence (in distribution) of the Markov chain was satisfied (Roberts and Rosenthal, 2007).

3. The full conditional for $\boldsymbol{\beta}$ has unnormalized density

$$[\boldsymbol{\beta}|\cdot] = [\boldsymbol{\beta}] \ \exp(-\Lambda(B)) \ (\pi_0 \, \Lambda(B))^{n_0} \prod_{i=1}^{n} \lambda(\boldsymbol{s}_i)$$

where $[\boldsymbol{\beta}]$ denotes the density function of a multivariate normal prior with mean $\mathbf{0}$ and diagonal covariance matrix $\sigma_\beta \boldsymbol{I}$. The scale parameter σ_β was assigned a value of 10 to specify an arbitrarily high level of prior uncertainty in the magnitude of $\boldsymbol{\beta}$. To sample the full conditional of $\boldsymbol{\beta}$, we used the approach described earlier (see item #2)

where $[\boldsymbol{\beta}|\cdot]$ is treated as the target density for samplers based on delayed-rejection, Metropolis-Hastings and adaptive Metropolis algorithms.

4. The full conditional for the parameters $\boldsymbol{\alpha}$, ξ, and σ has unnormalized density

$$[\boldsymbol{\alpha}, \xi, \sigma|\cdot] = [\boldsymbol{\alpha}][\xi][\sigma] \; (\pi_0 \, \Lambda(B))^{n_0} \prod_{i=1}^{n} \prod_{k=1}^{K} \exp[-\Phi(T_k, \boldsymbol{s}_i, \boldsymbol{x}_k)] \prod_{j=1}^{y_{ik}} \phi(t_{ikj}, \boldsymbol{s}_i, \boldsymbol{x}_k)$$

where $[\boldsymbol{\alpha}]$ denotes the density function of a multivariate normal prior with mean $\mathbf{0}$ and diagonal covariance matrix $\sigma_\alpha \boldsymbol{I}$. The scale parameter σ_α was assigned a value of 10 to specify an arbitrarily high level of prior uncertainty in the magnitude of $\boldsymbol{\alpha}$. Similarly, $[\xi]$ denotes the density function of a normal prior with mean zero and relatively high variance (10^2). A Half-t distribution with $\nu = 2$ degrees of freedom and scale parameter $s = 10$ was used to specify a weakly-informative prior for σ (Gelman, 2006); $[\sigma]$ denotes the density function of this prior. To sample the full conditional of $\boldsymbol{\alpha}$, ξ, and σ, we used the approach described earlier (see item #2) where $[\boldsymbol{\alpha}, \xi, \sigma|\cdot]$ is treated as the target density for samplers based on delayed-rejection, Metropolis-Hastings and adaptive Metropolis algorithms.

Restricted model of detection frequencies

We used a MCMC algorithm to generate a Markov chain whose stationary distribution is equivalent to a posterior with the following unnormalized density function:

$$[\boldsymbol{\theta}, n_0, \boldsymbol{s}_1, \ldots, \boldsymbol{s}_n | \boldsymbol{y}_{(1:n)}, n] \propto [\boldsymbol{\theta}][\boldsymbol{y}_{(1:n)}, n, n_0, \boldsymbol{s}_1, \ldots, \boldsymbol{s}_n | \boldsymbol{\theta}]$$

where $\boldsymbol{\theta} = (\boldsymbol{\beta}', \boldsymbol{\alpha}', \sigma)'$ denotes a vector of unknown parameters assumed to have mutually independent prior distributions (that is, $[\boldsymbol{\theta}] = [\boldsymbol{\beta}][\boldsymbol{\alpha}][\sigma]$). The posterior density function conditions on n, the number of distinct individuals observed during the sampling period, and on the frequencies of detection ($\boldsymbol{y}_{(1:n)}$) of these individuals.

As with the model of detection frequencies and detection times, we developed a MCMC algorithm that combined two sampling algorithms (delayed-rejection, Metropolis-Hastings (Tierney and Mira, 1999; Mira, 2001) and adaptive, Metropolis (Rosenthal, 2011)) to draw random samples from full conditional distributions. Except for parameter n_0, we sampled each full conditional using the approach described earlier (see item #2 above) where the full conditional density is treated as the target density for samplers based on delayed-rejection, Metropolis-Hastings and adaptive Metropolis algorithms. Therefore, for sake of brevity, below we simply describe the full conditional distributions sampled in one iteration of our MCMC algorithm:

1. The full conditional for n_0 has a familiar form: $n_0|\cdot \sim \text{Poisson}(\pi_0\,\Lambda(B))$, where

$$\pi_0\,\Lambda(B) = \int_B \lambda(\boldsymbol{s}) \prod_{k=1}^{K} \exp[-\Phi(T_k, \boldsymbol{s}, \boldsymbol{x}_k)]\,\mathrm{d}\boldsymbol{s}$$

(The integral required to compute $\pi_0\,\Lambda(B)$ cannot be evaluated in closed form. In practice this integral is approximated as a Riemann sum by partitioning B into a sufficiently fine grid.) The full conditional for n_0 is the conditional posterior for the number of activity centers of animals that were present in region B but not detected during the period of sampling. In this restriced model, $\Phi(T_k, \boldsymbol{s}, \boldsymbol{x}_k)$ can be expressed in closed form as follows:

$$\begin{aligned}\Phi(T_k, \boldsymbol{s}, \boldsymbol{x}_k) &= T_k\ \exp(\boldsymbol{\alpha}'\boldsymbol{w}_k - ||\boldsymbol{s} - \boldsymbol{x}_k||^2/(2\sigma^2)) \\ &= T_k\ \phi(\boldsymbol{s}, \boldsymbol{x}_k)\end{aligned}$$

2. The full conditional for $\boldsymbol{s}_i$ has unnormalized density

$$[\boldsymbol{s}_i|\cdot] = \lambda(\boldsymbol{s}_i) \prod_{k=1}^{K} \exp[-T_k\,\phi(\boldsymbol{s}_i, \boldsymbol{x}_k)]\ \phi(\boldsymbol{s}_i, \boldsymbol{x}_k)^{y_{ik}}$$

where $\phi(\boldsymbol{s}_i, \boldsymbol{x}_k) = \exp[\boldsymbol{\alpha}'\boldsymbol{w}_k - ||\boldsymbol{s}_i - \boldsymbol{x}_k||^2/(2\sigma^2)]$.

3. The full conditional for $\boldsymbol{\beta}$ has unnormalized density

$$[\boldsymbol{\beta}|\cdot] = [\boldsymbol{\beta}] \;\exp(-\Lambda(B))\; (\pi_0\, \Lambda(B))^{n_0} \prod_{i=1}^{n} \lambda(\boldsymbol{s}_i)$$

where $[\boldsymbol{\beta}]$ denotes the density function of a multivariate normal prior with mean $\boldsymbol{0}$ and diagonal covariance matrix $\sigma_\beta \boldsymbol{I}$. The scale parameter σ_β was assigned a value of 10 to specify an arbitrarily high level of prior uncertainty in the magnitude of $\boldsymbol{\beta}$.

4. The full conditional for the parameters $\boldsymbol{\alpha}$ and σ has unnormalized density

$$[\boldsymbol{\alpha}, \sigma|\cdot] = [\boldsymbol{\alpha}][\sigma] \;(\pi_0\, \Lambda(B))^{n_0} \prod_{i=1}^{n} \prod_{k=1}^{K} \exp[-T_k\, \phi(\boldsymbol{s}_i, \boldsymbol{x}_k)] \;\phi(\boldsymbol{s}_i, \boldsymbol{x}_k)^{y_{ik}}$$

where $[\boldsymbol{\alpha}]$ denotes the density function of a multivariate normal prior with mean $\boldsymbol{0}$ and diagonal covariance matrix $\sigma_\alpha \boldsymbol{I}$. The scale parameter σ_α was assigned a value of 10 to specify an arbitrarily high level of prior uncertainty in the magnitude of $\boldsymbol{\alpha}$. A Half-t distribution with $\nu = 2$ degrees of freedom and scale parameter $s = 10$ was used to specify a weakly-informative prior for σ (Gelman, 2006); $[\sigma]$ denotes the density function of this prior.

Posterior inference and estimation of Monte Carlo error

We used $M = 2000$ iterations of the MCMC algorithm to estimate summaries (means, standard deviations, quantiles) of the posterior distribution and other ecologically relevant functionals of the Markov chain. The estimates were computed using ergodic averages, which are simulation consistent (that is, the averages converge to posterior expectations as the number of iterations increases) according to the strong law of large numbers for Markov chains (Flegal and Jones, 2011). (The first 500 elements of the Markov chain were discarded to exclude initial transients in the Markov chain.) Monte Carlo standard errors of the

estimates were computed using the subsampling bootstrap method Flegal and Jones (2010, 2011) with overlapping batch means of size $\lfloor\sqrt{M}\rfloor$.

References

Flegal, J. M. and Jones, G. L. (2010). Batch means and spectral variance estimators in Markov chain Monte Carlo. *Annals of Statistics*, 38:1034–1070.

Flegal, J. M. and Jones, G. L. (2011). Implementing MCMC: estimating with confidence. In Brooks, S., Gelman, A., Jones, G. L., and Meng, X. L., editors, *Handbook of Markov chain Monte Carlo*, pages 175–197. Chapman & Hall / CRC, Boca Raton, Florida.

Gelfand, A. E. and Smith, A. F. M. (1990). Sampling-based approaches to calculating marginal densities. *Journal of the American Statistical Association*, 85:398–409.

Gelman, A. (2006). Prior distributions for variance parameters in hierarchical models (Comment on article by Browne and Draper). *Bayesian Analysis*, 1:515–534.

Mira, A. (2001). On Metropolis-Hastings algorithms with delayed rejection. *Metron*, 59:231–241.

R Core Team (2016). *R: A Language and Environment for Statistical Computing.* R Foundation for Statistical Computing, Vienna, Austria.

Roberts, G. O. and Rosenthal, J. S. (2007). Coupling and ergodicity of adaptive Markov chain Monte Carlo algorithms. *Journal of Applied Probability*, 44:458–475.

Rosenthal, J. S. (2011). Optimal proposal distributions and adaptive MCMC. In Brooks, S., Gelman, A., Jones, G. L., and Meng, X. L., editors, *Handbook of Markov chain Monte Carlo*, pages 93–111. Chapman & Hall / CRC, Boca Raton, Florida.

Tierney, L. and Mira, A. (1999). Some adaptive Monte Carlo methods for Bayesian inference. *Statistics in Medicine*, 18:2507–2515.

Disclaimers

The computing program (R Core Team, 2016) needed to implement our MCMC algorithm is available in S4 Appendix.[2] Any use of trade, firm, or product names is for descriptive purposes only and does not imply endorsement by the U.S. Government.

[2]This software has been approved for release by the U.S. Geological Survey (USGS). Although the software has been subjected to rigorous review, the USGS reserves the right to update the software as needed pursuant to further analysis and review. No warranty, expressed or implied, is made by the USGS or the U.S. Government as to the functionality of the software and related material nor shall the fact of release constitute any such warranty. Furthermore, the software is released on condition that neither the USGS nor the U.S. Government shall be held liable for any damages resulting from its authorized or unauthorized use.
