# Supplementary material for: A hierarchical model for estimating the spatial distribution and abundance of animals detected by continuous-time recorders
Source: PLoS One. 2017 May 17;12(5):e0176966. doi: 10.1371/journal.pone.0176966 (PMC5435310; doi:10.1371/journal.pone.0176966)
Supplement: S3 Appendix — (PDF) [file pone.0176966.s003.pdf]

S3 Appendix: Description of spatial covariates used in simulation study[1]

In this supplement we describe how the values of the two spatial covariates used in the simulation study were computed.

Spatial covariate $v(\boldsymbol{s})$ of individual density

We computed $v(\boldsymbol{s})$ using the probability density function of a finite mixture of four normal (Gaussian) distributions as follows:

$$v(\boldsymbol{s}) = \sum_{i=1}^{4} p_i \, f(\boldsymbol{s}|\boldsymbol{\mu}_i, \boldsymbol{\Sigma}_i)$$

where the mixing proportions were $p_1 = 0.25$, $p_2 = 0.45$, $p_3 = 0.15$, and $p_4 = 0.15$. The means and covariance matrices for the four normal distributions were as follows:

- $\boldsymbol{\mu}_1 = \begin{pmatrix} 0.75 \\ -0.2 \end{pmatrix}, \quad \boldsymbol{\Sigma}_1 = \begin{pmatrix} 0.25 & 0.25 \\ 0.25 & 1.00 \end{pmatrix}$
- $\boldsymbol{\mu}_2 = \begin{pmatrix} -0.7 \\ 0.6 \end{pmatrix}, \quad \boldsymbol{\Sigma}_2 = \begin{pmatrix} 1.00 & -0.2 \\ -0.2 & 0.25 \end{pmatrix}$
- $\boldsymbol{\mu}_3 = \begin{pmatrix} -1.5 \\ -1.0 \end{pmatrix}, \quad \boldsymbol{\Sigma}_3 = \begin{pmatrix} 0.15 & 0.1 \\ 0.1 & 0.25 \end{pmatrix}$
- $\boldsymbol{\mu}_4 = \begin{pmatrix} 1.75 \\ 1.75 \end{pmatrix}, \quad \boldsymbol{\Sigma}_4 = \begin{pmatrix} 0.15 & -0.1 \\ -0.1 & 0.25 \end{pmatrix}$

[1] This draft manuscript is distributed solely for purposes of scientific peer review. Its content is deliberative and predecisional, so it must not be disclosed or released by reviewers. Because the manuscript has not yet been approved for publication by the U.S. Geological Survey (USGS), it does not represent any official USGS finding or policy.

After $v(\boldsymbol{s})$ was computed for all locations $\boldsymbol{s} \in B$, the values of $v(\boldsymbol{s})$ were centered and scaled to have zero mean and unit variance.

Spatial covariate $w(\boldsymbol{s})$ of baseline detection rate

We computed $w(\boldsymbol{s})$ as a spatial trend (decline) in the northeast direction as follows:

$$w(\boldsymbol{s}) = -0.1s_1 - 0.1s_2 + 0.01s_1s_2$$

where s_1 = easting of location $\boldsymbol{s}$ and s_2 = northing of location $\boldsymbol{s}$. After $w(\boldsymbol{s})$ was computed for all K trap locations (i.e., where $\boldsymbol{s} = \boldsymbol{x}_k$), the values of $w(\boldsymbol{s})$ were centered and scaled to have zero mean and unit variance.
